# Combined chemotherapy plus endostar with sequential stereotactic radiotherapy as salvage treatment for recurrent esophageal cancer with severe dyspnea: A case report and review of the literature

**DOI:** 10.3892/ol.2014.2087

**Published:** 2014-04-25

**Authors:** MINGFANG XU, HUAN HUANG, YANLI XIONG, BO PENG, ZEJUN ZHOU, DONG WANG, XUEQIN YANG

**Affiliations:** Cancer Center, Daping Hospital, Third Military Medical University, Chongqing 400042, P.R. China

**Keywords:** esophageal cancer, stereotactic radiotherapy, chemotherapy, endostar

## Abstract

For the majority of inoperable esophageal cancer cases, chemoradiotherapy is the most suitable treatment option. Cetuximab may provide certain benefits, however, this can be an expensive therapy. Additionally, stereotactic body radiation therapy (SBRT) is typically contraindicated for esophageal cancer due to the potential for esophageal perforation and stenosis. The use of combined chemotherapy plus endostar with sequential SBRT for the treatment of esophageal squamous cancer has not been reported. In the current study, the case of a 71-year-old female with esophageal squamous cancer diagnosed 2 years prior is presented. Surgery and four cycles of cisplatin plus 5-fluorouracil chemotherapy had been administered. The patient showed recurrence at the paratracheal lymph node, exhibited severe dyspnea (grade III) and required a semi-liquid diet. Four cycles of the docetaxel, 5-fluorouracil and nedaplatin regimen plus endostar (3 mg; days 1–14; intravenously) with sequential SBRT (3300 cGy in 10 fractions) was administered. Following treatment, the symptoms of the patient completely disappeared, and objective efficacy evaluation indicated complete remission. At the time of writing, the patient is living without discomfort and the progression-free survival is >8 months. In conclusion, the present case indicates that combined treatment of chemotherapy and endostar with sequential stereotactic radiotherapy is a safe and effective option for the management of esophageal cancer.

## Introduction

Although esophageal cancer is not as common as lung, breast or colon malignancies, it is associated with a high mortality rate, with an incidence rate that is close to its cancer-specific mortality (1). Due to presentation with severe comorbidity and an advanced stage of disease, over half of patients with esophageal cancer in the Western world are not able to undergo surgery (2). The 5-year survival rate for resectable disease ranges from 10–25% (3), while recurrent and metastatic esophageal cancer have even poorer prognoses. Progression-free survival for esophageal cancer is reportedly 5.6 months (95 % confidence interval [CI], 2.8–8.4 months), with a median survival time of 17.0 months (95 % CI, 12.3–21.7 months) (4).

The main aim when treating patients with inoperable esophageal cancer is to sustain oral nutrition. Minimizing the hospital stay, relieving pain and eliminating reflux and regurgitation are also essential, and therefore treatment is modified to the individual depending on the tumor stage and location, and the overall health of the patient. Although combination chemoradiotherapy has been associated with a high rate of complete pathological remission (5), the majority of targeted drugs, including avastin and cetuximab (6–7), have limited roles in the treatment of esophageal squamous cancer. While cetuximab may provide certain benefits in the treatment of this type of cancer (7), it is an expensive treatment. These observations underline the requirement for exploration of other targeted drugs for the treatment of esophageal squamous cancer.

Radiotherapy together with chemotherapy and surgery represents the main treatment modality for esophageal cancer. However, technological advances in radiation treatment, including positron emission tomography-based, planning intensity-modulated (IMRT) and image-guided radiotherapies, have developed the practice, particularly for the treatment of esophageal cancer. The main goal of these novel approaches is precise irradiation of the tumor, while minimizing the risk of damage to healthy tissues (8). Post-treatment complications are often associated with esophageal cancer radiotherapy and patients have to endure radiotherapy for long periods of time. In addition, certain patients present with general radiotherapy contraindications, including chronic obstructive pulmonary disease (COPD) and cardiopathy. Although stereotactic body radiation therapy (SBRT) is increasingly used to treat numerous solid tumors, it is usually prohibited for use in the treatment of esophageal cancer due to the potential for esophageal perforation and stenosis. Thus, more efficient management should be explored to improve survival and quality of life for patients with advanced esophageal cancer.

In the present study, a combination of chemotherapy and recombinant human endostatin (endostar) with sequential SBRT is shown to be successful as a salvage treatment for recurrent esophageal cancer accompanied by grade III dyspnea and difficulty in swallowing a semi-liquid diet. The patient had a relatively long progression-free survival time of >8 months. Patient provided written informed consent. This study was approved by the ethics committee of Daping Hospital (Chongqing, China).

## Case report

**Figure 1:**
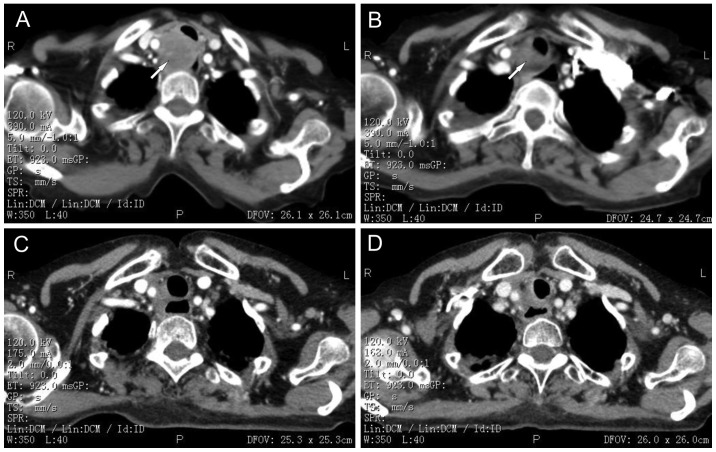
Enhanced computed tomography (CT) scans of the patient during treatment. (A) Enhanced CT scan demonstrating a paratracheal lymph node metastasis, 4.5×3.5×2.5 cm (white arrow), squeezing the trachea and esophagus. (B) Enhanced CT scan demonstrating the lesion reduced in size to 3.0×2.5×1.5 cm (white arrow) 1 month following a cycle of chemotherapy combined with endostar. Objective evaluation indicated partial remission. (C) Enhanced CT scan 1 month following stereotactic radiotherapy treatment. Objective evaluation indicated near complete remission. (D) Enhanced CT scan 4 months following radiotherapy and four cycles of chemotherapy combined with endostar. Objective evaluation indicated complete remission.

A 71-year-old female with a 2-year history of esophageal squamous cell carcinoma was referred to the Cancer Center of Daping Hospital (Chongqing, China). The patient had severe grade III dyspnea for 6 days and required a semi-liquid diet for 2 years. The performance status (PS) upon admission was 4 points according to the Eastern Cooperative Oncology Group scale. The medical history of the patient included COPD, diagnosed in 1999, and diabetes, diagnosed in 2008, with no history of smoking or drinking. The patient had undergone surgery 2 years prior, followed by four cycles of cisplatin plus 5-fluorouracil as a post-operative adjuvant therapy. At the time of the initial surgery, the cancer was classified as T2N0M0 stage. A computed tomography (CT) of the chest (Fig. 1A) prior to admission revealed the presence of a paratracheal lymph node metastasis, measuring 4.5×3.5×2.5 cm, that was squeezing the trachea and esophagus. No other metastasis in the abdominal organs or lymph nodes were detected by CT.

Upon referral, the airway of the patient was so narrow that endotracheal intubation was unsuccessful and, consequently, the patient was unsuitable for tracheotomy due to the location of the stenosis. For similar reasons, the patient was also unsuitable for surgical resection. In addition, dyspnea is a contraindication for radiotherapy, as radiation edema may aggravate the condition. As the life of the patient was in danger, methylprednisolone acetate was administered at 80 mg per day for temporary relief of the dyspnea, and this was followed by initial treatment with chemotherapy combined with endostar. As the PS of the patient was 4, the chemotherapy regimen, which included intravenous (IV) administration of docetaxel (40 mg/m^2^; days 1 and 8), 5-fluorouracil (400 mg/m^2^; days 1–5 and 8–12), nedaplatin (40 mg/m^2^; days 1 and 8) and endostar (3 mg; days 1–14), was divided into 2 weeks. The dyspnea of the patient was downgraded to grade II after 1 week, and the methylprednisolone acetate dose was reduced to 40 mg per day. One month following chemotherapy, the dyspnea of the patient was alleviated. The dyspnea was downgraded to grade I and a CT scan showed that the lesions were 3.0×2.5×1.5 cm (Fig. 1B). Objective efficacy evaluation indicated partial remission.

The patient was then administered SBRT treatment combined with methylprednisolone acetate at 40 mg per day until the end of radiotherapy. The stereotactic γ-ray whole-body therapeutic system (body γ-knife radiosurgery) with 30 rotary conical-surface Co (60) sources was focused on the target volume. Low-speed CT simulation was conducted, followed by three-dimensional conformal radiotherapy (3D-CRT) planning. A total dose of 33 Gy was delivered at 3.3 Gy/fraction to the 60% isodose line covering the planning target volume. The biological equivalent dose (BED) was 43.89 Gy. The radiotherapy course was delivered in 2 weeks. During the radiotherapy, the patient experienced grade II esophagitis. One month following radiotherapy, the patient showed rapid improvement in dyspnea and dysphagia, and CT revealed that the paratracheal lymph node lesions had disappeared (Fig. 1C). Objective efficacy evaluation indicated almost complete remission.

The patient then received another three cycles (separated by a 3-week interval) of IV systemic chemotherapy combined with endostar (docetaxel: 75 mg/m^2^, day 1; 5-fluorouracil: 800 mg/m^2^, days 1–5; nedaplatin: 75 mg/m^2^, day 1; and endostar: 3 mg; days 1–14). Four months following radiotherapy, CT imaging revealed that the cancer was in complete remission (Fig. 1D). The patient was virtually asymptomatic following this treatment, and was able to breathe, eat normally and gain weight. Significantly, radiation-induced esophageal perforation and stenosis was not observed. The patient continued to exhibit no symptoms of dyspnea and dysphagia and has had no evidence of metastatic disease, with a progression-free survival of >8 months at the time of writing.

## Discussion

In the present study, the value of SBRT in patients with esophageal cancer, who are not suitable for surgery due to the advanced disease stage or comorbidity was assessed. The case of a patient with esophageal cancer who responded to SBRT treatment is presented, including the use of combined chemotherapy plus endostar with sequential SBRT for the treatment of esophageal squamous cancer.

Esophageal carcinoma is one of the most common malignant tumors, particularly in China, which is a high-incidence area. Due to the mild symptoms associated with early-stage esophageal cancer, the majority of patients cannot be diagnosed until they progress to advanced cancer. The treatment outcomes for surgery or chemoradiotherapy for advanced-stage patients remain unsatisfactory (9). Although esophagectomy has historically been considered the standard of care, ~60% of patients are unsuitable for surgical resection due to advanced-stage disease or the presence of comorbidity (10). This involves the infiltration of surrounding tissues by the esophageal cancer, causing esophageal stenosis and possibly esophagotracheal fistula in certain cases. The median survival time for patients with advanced esophageal cancer in a case-control study was only 3–5 months (11). These patients can only be treated with palliative procedures, including radiotherapy, chemotherapy and esophageal stent placement, to improve their quality of life. The standard scheme for concurrent chemoradiotherapy is radiotherapy at a dose of 50–50.4 Gy over 5–5.5 weeks and 5-fluorouracil and cisplatin-based concurrent chemotherapy (12–13).

Radiotherapy methods for treatment of esophageal cancer are 3D-CRT, IMRT and afterloading intracavitary radiotherapy. Although the current worldwide standard of esophageal cancer radiation treatment is 3D-CRT, overall survival, locoregional control and non-cancer-related mortality were significantly improved following IMRT compared with those following 3D-CRT (14). Nutting *et al* (15) concluded that IMRT using conventional beam angles can provide acceptable dose homogeneity within the planned target volume and reduce lung irradiation compared to 3D-CRT. Additionally, numerous studies have revealed the benefits of IMRT in various tumor sites in terms of the feasibility of normal tissue sparing (16–18). Postponement complications for esophageal cancer radiotherapy include esophagitis and toxicity of the lung, heart and spinal cord, while patients with severe comorbidity, advanced age and advanced-stage disease cannot endure the long treatment time and are not suitable for IMRT. Afterloading intracavitary radiotherapy offers effective palliation as a method of brachytherapy, but is also associated with multiple endoscopies, transient acute mucosal edema causing symptom exacerbation and an increased risk of esophageal fistula formation (19–20). Localized toxicity may be the consequence of difficult catheter-luminal centering and the resulting poor dosimetry of high-dose rate brachytherapy. Additionally, brachytherapy is often complicated by the difficulty in confirmation of the final catheter position (21).

SBRT has been accepted as an effective cancer therapy by patients and physicians. The goal of SBRT is precise, and complete destruction of chosen target structures without significant concomitant or late-radiation damage to adjacent tissues. This effect is obtained by the precise focusing of multiple low-energy radiation beams crossing at the target, operating by the radiobiological effect of stereotactically directed, highly focused ionizing γ-beams of the 201 cobalt-60 sources. The mechanical accuracy is ~0.3 mm, which was originally developed primarily to treat brain tumors, but was soon used for the treatment of other tumors.

Although tumor location in the cavity is usually prohibitive for SBRT treatment, radiation esophagitis was slight in the present study, and radiation-induced esophageal perforation and stenosis were not observed. The dose of only 33 Gy (BED, 43.89 Gy) used in the present study was able to achieve local control and provide symptom relief. These findings indicate that tumor invasion of the whole layer of the esophagus, and not SBRT, is the primary factor responsible for radiation-induced esophageal perforation, and everyday esophageal movement itself can prevent esophageal stenosis. Thus, if the single-dose radiation is not too high, esophagus cancer is not an absolute contraindication to SBRT treatment. Additionally, although SBRT is not a standard treatment option for esophageal cancer, it appears to be a reasonable option in patients who have become refractory to traditional therapy, and it can rapidly relieve patient symptoms when administered at the appropriate dose. More specifically, SBRT is a good treatment option for certain cases with severe comorbidity, advanced age and advanced disease stage. However, further clinical trials are required to explore the safety and efficacy of SBRT for the treatment of esophageal cancer, particularly with regard to late complications.

SBRT was combined with endostar, a proteolytic C-terminal fragment of the vascular and epithelial basement membrane collagen type XVIII that has been proven to be effective for anti-angiogenesis and tumor growth inhibition (22), to treat the patient with esophageal cancer in the present study. Endostar combined with radiation or chemotherapy has proven effective in treating other cancers, including nasopharyngeal carcinoma, colorectal, non-small cell lung and cervical cancers (23–25). In addition, Chang *et al* (26) investigated the efficacy of endostar combined with chemotherapy on human esophageal squamous cell carcinoma Eca-109 cells in mice, and demonstrated that the tumor volumes and weight were significantly lower in the treatment group compared with the control. The cellular proliferation of the tumor xenograft in the combined treatment group was significantly lower. These findings indicate that endostar combined with chemotherapy can evidently enhance the inhibitory effect on esophageal squamous cell carcinoma Eca-109 cells in mice. A review of the outcomes of endostar combined with chemoradiotherapy as a first-line treatment for patients with unresectable esophageal cancer showed that chemoradiotherapy combined with endostatin increased the complete response rate (44.4 vs. 30% in the chemoradiotherapy-alone group) and the 1-year and 3-year overall survival rates (72 vs. 50.0% and 32 vs. 22.0%, respectively) (27). Although cetuximab combined with chemotherapy is also effective in the treatment of esophageal squamous cell cancer according to the National Comprehensive Cancer Network guidelines (9), endostar combined with chemotherapy was chosen for the present study due to economic reasons. The results show that this regimen was effective, safe and reversed the development of the disease with the initial treatment. In addition, endostar prolonged the progression-free survival of the patient. However, further clinical trials are required to confirm its role in the treatment of esophageal cancer.

## References

[1] Karaosmanoğlu AD, Blake MA (2012). Applications of PET-CT in patients with esophageal cancer. Diagn Interv Radiol.

[2] Koshy M, Esiashvilli N, Landry JC (2004). Multiple management modalities in esophageal cancer: epidemiology, presentation and progression, work-up, and surgical approaches. Oncologist.

[3] Tytgat GN, Bartelink H, Bernards R (2004). Cancer of the esophagus and gastric cardia: recent advances. Dis Esophagus.

[4] Huang J, Zhou Y, Zhang H (2013). A phase II study of biweekly paclitaxel and cisplatin chemotherapy for recurrent or metastatic esophageal squamous cell carcinoma: ERCC1 expression predicts response to chemotherapy. Med Oncol.

[5] Emi M, Hihara J, Hamai Y (2012). Neoadjuvant chemoradiotherapy with docetaxel, cisplatin, and 5-fluorouracil for esophageal cancer. Cancer Chemother Pharmacol.

[6] Idelevich E, Kashtan H, Klein Y (2012). Prospective phase II study of neoadjuvant therapy with cisplatin, 5-fluorouracil, and bevacizumab for locally advanced resectable esophageal cancer. Onkologie.

[7] Lorenzen S, Schuster T, Porschen R (2009). Cetuximab plus cisplatin-5-fluorouracil versus cisplatin-5-fluorouracil alone in first-line metastatic squamous cell carcinoma of the esophagus: a randomized phase II study of the Arbeitsgemeinschaft Internistische Onkologie. Ann Oncol.

[8] Vosmik M, Petera J, Sirak I (2010). Technological advances in radiotherapy for esophageal cancer. World J Gastroenterol.

[9] Gao XS (2010). Considerations of treatment standardization from the procession of NCCN guideline of esophageal cancer. Chin J Cancer.

[10] Katsoulis IE, Karoon A, Mylvaganam S, Livingstone JI (2006). Endoscopic palliation of malignant dysphagia: a challenging task in inoperable oesophageal cancer. World J Surg Oncol.

[11] Wong SK, Chiu PW, Leung SF (2008). Concurrent chemoradiotherapy or endoscopic stenting for advanced squamous cell carcinoma of esophagus: a case-control study. Ann Surg Oncol.

[12] Cooper JS, Guo MD, Herskovic A, Radiation Therapy Oncology Group (1999). Chemoradiotherapy of locally advanced esophageal cancer: long-term follow-up of a prospective randomized trial (RTOG 85-01). JAMA.

[13] Minsky BD, Pajak TF, Ginsberg RJ (2002). INT 0123 (Radiation Therapy Oncology Group 94-05) phase III trial of combined-modality therapy for esophageal cancer: high-dose versus standard-dose radiation therapy. J Clin Oncol.

[14] Lin SH, Wang L, Myles B (2012). Propensity score-based comparison of long-term outcomes with 3-dimensional conformal radiotherapy vs intensity-modulated radiotherapy for esophageal cancer. Int J Radiat Oncol Biol Phys.

[15] Nutting CM, Bedford JL, Cosgrove VP (2001). A comparison of conformal and intensity-modulated techniques for oesophageal radiotherapy. Radiother Oncol.

[16] Mundt AJ, Mell LK, Roeske JC (2003). Preliminary analysis of chronic gastrointestinal toxicity in gynecology patients treated with intensity-modulated whole pelvic radiation therapy. Int J Radiat Oncol Biol Phys.

[17] Uy NW, Woo SY, Teh BS (2003). Intensity-modulated radiation therapy (IMRT) for meningioma. Int J Radiat Oncol Biol Phys.

[18] Vicini FA, Sharpe M, Kestin L (2002). Optimizing breast cancer treatment efficacy with intensity-modulated radiotherapy. Int J Radiat Oncol Biol Phys.

[19] Homs MY, Eijkenboom WM, Coen VL (2003). High dose rate brachytherapy for the palliation of malignant dysphagia. Radiother Oncol.

[20] Homs MY, Steyerberg EW, Eijkenboom WM (2004). Single-dose brachytherapy versus metal stent placement for the palliation of dysphagia from oesophageal cancer: multicentre randomised trial. Lancet.

[21] Russo JK, Rosen L (2011). TomoTherapy stereotactic body radiation therapy (SBRT) for the salvage treatment of locally recurrent esophageal adenocarcinoma following trimodality therapy: a case report. Tumori.

[22] O’Reilly MS, Boehm T, Shing Y (1997). Endostatin: an endogenous inhibitor of angiogenesis and tumor growth. Cell.

[23] Rong B, Yang S, Li W (2012). Systematic review and meta-analysis of Endostar (rh-endostatin) combined with chemotherapy versus chemotherapy alone for treating advanced non-small cell lung cancer. World J Surg Oncol.

[24] Zhou J, Wang L, Xu X (2012). Antitumor activity of Endostar combined with radiation against human nasopharyngeal carcinoma in mouse xenograft models. Oncol Lett.

[25] Jin F, Ji H, Jia C (2012). Synergistic antitumor effects of endostar in combination with oxaliplatin via inhibition of HIF and CXCR4 in the colorectal cell line SW1116. PLoS One.

[26] Chang L, Guo F, Lv Y (2013). The inhibitory effects of Endostar combined with chemotherapy on human esophageal squamous cell carcinoma xenograft in mice. Mol Biol Rep.

[27] Zhong Z, Gu X, Zhang Z (2012). Recombinant human endostatin combined with definitive chemoradiotherapy as primary treatment for patients with unresectable but without systemic metastatic squamous cell carcinoma of the oesophagus. Br J Radiol.

